# β1-Syntrophin Modulation by miR-222 in *mdx* Mice

**DOI:** 10.1371/journal.pone.0012098

**Published:** 2010-08-10

**Authors:** Valeria De Arcangelis, Filippo Serra, Carlo Cogoni, Elisabetta Vivarelli, Lucia Monaco, Fabio Naro

**Affiliations:** 1 Department of Histology and Medical Embryology, University Sapienza, Rome, Italy; 2 Department of Physiology and Pharmacology, University Sapienza, Rome, Italy; 3 Department of Cellular Biotechnology and Ematology, University Sapienza, Rome, Italy; 4 IIM, Pavia, Italy; Hospital 12 Octubre Madrid, Spain

## Abstract

**Background:**

In *mdx* mice, the absence of dystrophin leads to the deficiency of other components of the dystrophin-glycoprotein complex (DAPC), making skeletal muscle fibers more susceptible to necrosis. The mechanisms involved in the disappearance of the DAPC are not completely understood. The muscles of *mdx* mice express normal amounts of mRNA for the DAPC components, thus suggesting post-transcriptional regulation.

**Methodology/Principal Findings:**

We investigated the hypothesis that DAPC reduction could be associated with the microRNA system. Among the possible microRNAs (miRs) found to be upregulated in the skeletal muscle tissue of *mdx* compared to wt mice, we demonstrated that miR-222 specifically binds to the 3′-UTR of β1-syntrophin and participates in the downregulation of β1-syntrophin. In addition, we documented an altered regulation of the 3′-UTR of β1-syntrophin in muscle tissue from dystrophic mice.

**Conclusion/Significance:**

These results show the importance of the microRNA system in the regulation of DAPC components in dystrophic muscle, and suggest a potential role of miRs in the pathophysiology of dystrophy.

## Introduction

Dystrophin is a cytoplasmic protein belonging to a large oligomeric complex (dystrophin-associated protein complex, or DAPC) that associates with other proteins, including sarcoglycans, syntrophins, dystroglycans, syntrobrevin, and sarcospan [1]–[3]. The DAPC plays an important structural role in linking the actin cytoskeleton to the extracellular matrix. A disruption of the DAPC impairs muscle fiber function; therefore, several myopathies are due to genetic defects found in the DAPC proteins [3]–[11]. The most common myopathies caused by a defect in the DAPC complex are Duchenne Muscular Dystrophy (DMD) and its milder form, Becker Muscular Dystrophy, which are both caused by mutations in the X−linked dystrophin gene [4]–[6]. Defects in sarcoglycan (SG) subunits are associated with an autosomal recessive form of Limb Girdle Muscular Dystrophy [7], [8]. Although no human diseases have been found to result from mutations in the dystroglycan gene, impaired glycosylation of the α-dystroglycan subunit due to defects in glycosyltransferases leads to muscular disorders [9], [10]. A deficiency of the syntrophin-dystrobrevin subcomplex has been observed in patients with inherited myopathy [11].

Different animal models are available to study the different dystrophies. The most commonly used laboratory animal model of DMD is the *mdx* mouse. In these animals, all the muscles lack dystrophin, however, *mdx* mice show a much milder phenotype than DMD patients [12]. Although the muscles of *mdx* mice are affected to a different extent, physical exercise worsens the pathology, similar to that observed in the human disease [13]. Genetically modified animal models that are deficient in the four different SG subunits have also been developed, and their phenotype is associated with skeletal and cardiac myopathies [14]–[18].

Studies using these animal models of different myopathies revealed that the DAPC is tightly regulated. A deletion or mutation in the gene of one of the components of the DAPC leads to destabilization of the entire complex and a strong reduction in the intracellular concentration of the other proteins [14]–[19]. The mechanisms involved in this phenomenon are not yet completely understood. Because treatment with proteasome inhibitors, have been shown to promote upregulation of the expression levels of some members of the DAPC in *mdx* mice and in the muscular explants obtained from patients with DMD or BMD, it has been suggested that the degradation system is involved in inhibiting DAPC proteins expression in dystrophy [20]–[22].

In recent years, mounting evidence has shown the pivotal role of small, non-coding RNAs, such as microRNAs (miRs), in the negative regulation of gene expression [23]–[25]. In the nucleus, miRs are transcribed as long primary transcripts (pri-miRs) and processed into 60–120 nucleotide hairpin precursors (pre-miRs), which are exported to the cytoplasm where they are further processed into mature 21–23 nucleotide transcripts. One of the two strands of the mature microRNAs is incorporated into the large protein complex, RISC (RNA-Induced Silencing Complex), and guides the complex to the target mRNA. MicroRNA modulation of gene expression can occur by blocking translation or by cleavage and degradation of the target mRNA [23]–[25]. Several miRs (miR-1, miR-133, and miR-206) have been shown to be specifically expressed in the skeletal muscle [26]–[32]. These miRs could play a role in numerous muscular diseases, as microarray analysis of muscle samples obtained from patients affected by different muscular disorders, including DMD and BMD, revealed that approximately 200 miRs were differentially expressed [30], [31]. Among these miRs, five (miR-146b, miR-221, miR-155, miR-214, and miR-222) were found to be consistently dysregulated in the different analyzed diseases [31]. In a recent study, Greco et al. [32] reported the detection of a common miRNA signature in muscles from *mdx* mice and DMD patients. Here, 11 miRs were found to be dysregulated in both types of samples and were suggested to be involved in the pathways implicated in the response to muscle damage.

To date, no information is available regarding the function of these dysregulated miRs in the different myopathies. We addressed the potential role of miRs in the pathogenesis of DMD, considering that, in muscle tissue obtained from *mdx* and transgenic mice (α-and β-sarcoglycan knockout mice) normal levels of mRNA for the different components of the DAPC were detected, in spite of the absence of the corresponding proteins, thus ruling out transcriptional regulation of the specific mRNAs [14]–[19]. We verified the possible involvement of the microRNA system as a regulator of the DAPC proteins using the *mdx* mouse model of DMD. We analyzed both mRNA and protein levels of syntrophins and dystroglycans, and focused our study on evaluating the regulation of β1-syntrophin. By analyzing the 3′ untranslated region (3′UTR) of β1-syntrophin, we found that three miRs could target this protein, and we established that one of these, miR-222, is upregulated in the muscles of *mdx* mice and is involved in the downregulation of the β1-syntrophin isoform in dystrophic muscles.

## Results

### RNA and protein expression levels

It has been previously reported that when one of the DAPC components is genetically absent, other proteins of the complex are likewise reduced, and complex formation is ultimately impaired. To confirm this phenomenon, we monitored the mRNA levels, protein expression and immunolocalization of four DAPC components (α- and β-dystroglycan and α, and β1-syntrophin) in different muscles (gastrocnemius, tibialis, extensor digitorum longus, diaphragm) obtained from normal and *mdx* dystrophic mice.

**Figure 1:**
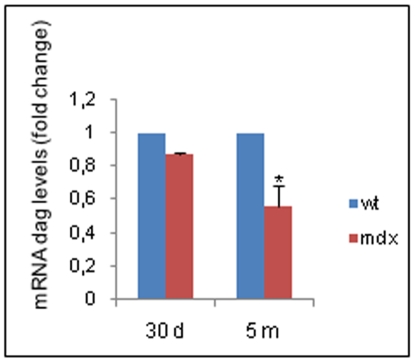
Dystroglycan mRNA expression. The mRNA levels from the gastrocnemius muscle tissues of wt and *mdx* mice of different ages (30 d, 30-day-old mice; 5 m: five-month-old mice) were assessed by qRT-PCR; relative gene expression was calculated by the comparative Ct method (2^−ddCt^). The Ct values of each gene were normalized to the Ct value of GAPDH in the same RNA sample. The mRNA levels in the *mdx* samples are expressed as fold change compared to those in wt samples. All values represent the mean ± SD from experiments performed on three different RNA preparations of the muscle tissues from wt and *mdx* mice (see Methods). *P<0,05 versus wt (*P* = 0,0465 measured by the Mann-Whitney test).

The dystroglycan gene (*Dag*) encodes for a single polypeptide that is post-translationally cleaved into two subunits, α-dystroglycan (α-dag) and β-dystroglycan (β-dag) [33]. Using quantitative real time RT-PCR (qRT-PCR), a slight, but not significant decrease in *dag* mRNA level, was observed in the gastrocnemius muscles of young *mdx* mice compared to those of wild type (wt) animals. With increasing age, a 50% decrease in *dag* mRNA level was observed in the dystrophic muscles compared to the muscles of wt adult animals (Fig. 1). Although the protein level of the α-dag subunit was not statistically different between muscle tissues from wt and *mdx* animals of the same age, α-dag was not correctly localized at the sarcolemma of dystrophic muscle cells (Fig. S1A). The protein level of β-dag tended to decrease in both young and adult muscle tissues from dystrophic mice (Fig. S1B); similar to that of the α-subunit, localization of the β-dag protein at the sarcolemma was clearly impaired in the muscle tissues from dystrophic mice (Fig. S1C). Taken together, these data suggest that a small decrease in α- and β-dystroglycan protein levels occurs in the gastrocnemius muscles of *mdx* dystrophic animals, probably due to a reduction in *Dag* gene transcription.

**Figure 2:**
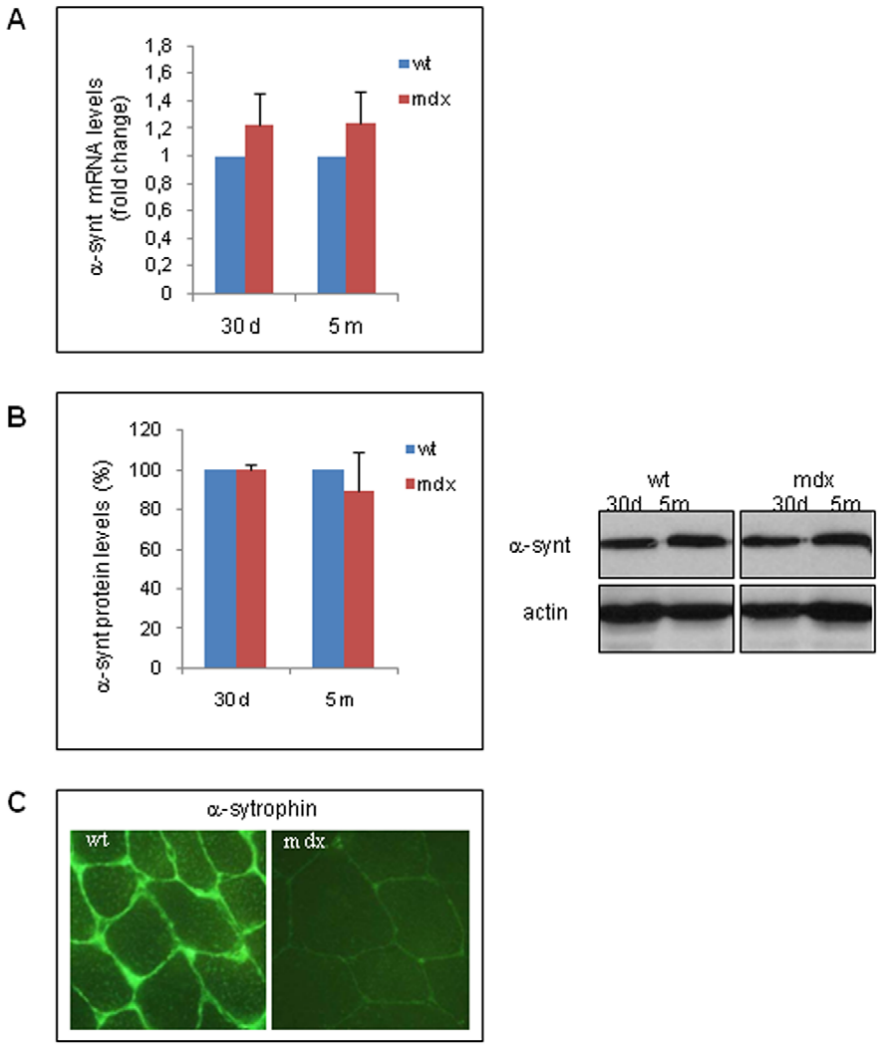
α-syntrophin mRNA and protein expression. A: The mRNA levels in the gastrocnemius muscle tissues from wt and *mdx* mice of different ages (30 d, 30-day-old mice; 5 m: five-month-old mice) were determined by qRT-PCR and calculated by the comparative Ct method (2^−ddCt^). The Ct values of each gene are normalized to the Ct value of GAPDH in the same RNA sample. The mRNA levels in the *mdx* samples are expressed as fold changes compared to those in wt samples. All values represent the mean ± SD from three different experiments performed on different RNA preparations of the muscle tissues from wt and *mdx* mice (see Methods). B: Total protein extracts from the gastrocnemius muscle tissues of wt and *mdx* mice of the indicated ages were resolved by SDS-PAGE and probed with an α-syntrophin antibody. A representative western blot is shown. The graph values represent the mean ± SD of the densitometric analyses from three different experiments with different animal samples (see Methods). Data are presented as the percentage of protein in *mdx* mice compared to that in wt mice, normalized to endogenous actin expression level. C: Frozen sections of the gastrocnemius muscles obtained from wt and *mdx* adult mice were probed with an anti-α-syntrophin antibody and visualized using a secondary antibody coupled to a fluorescent marker, FITC. A representative of the two performed analyses on two different five-months-old wt and *mdx* mice, is shown.

Among the different syntrophin isoforms expressed in skeletal muscle tissues, the α and β1 subunits are preferentially associated with dystrophin and are encoded by two different genes [34]. The mRNA level of the α isoform of syntrophin (α-synt) was slightly increased in the gastrocnemius muscles of *mdx* mice (Fig. 2A). There was no difference in α-syntrophin protein levels between the normal and *mdx* animals; however, the protein was no longer localized at the plasma membrane of dystrophic muscle fibers, shown by immunofluorescence analysis (Figs. 2B and 2C). These observations suggest that the absence of α-syntrophin at the cell membrane is probably related to the absence of the entire DAPC but not due to a reduction in its protein levels.

**Figure 3:**
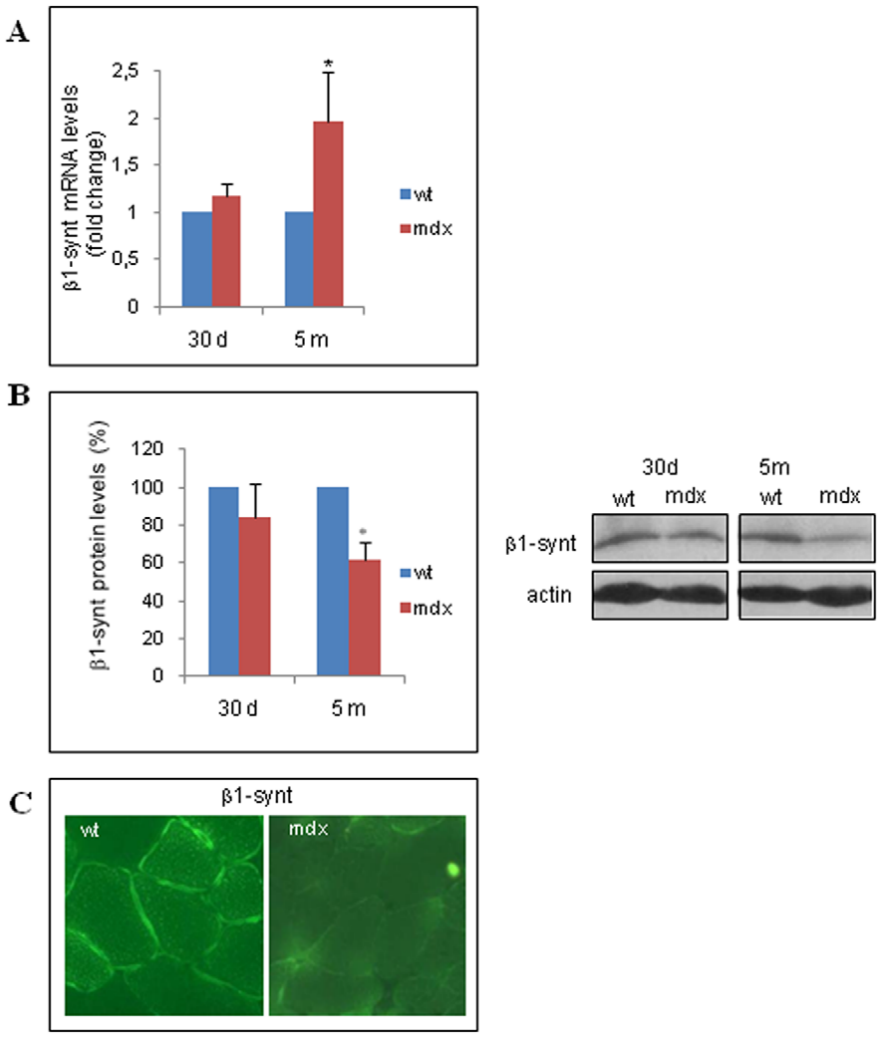
β1-syntrophin mRNA and protein expression. A: The mRNA levels in from the gastrocnemius muscle tissues of wt and *mdx* mice of different ages (30 d, 30-day-old mice; 5 m: five-month-old mice) were determined by qRT-PCR and calculated by the comparative Ct method (2^−ddCt^). The Ct values from each gene are normalized to the Ct value of GAPDH in the same RNA sample. The mRNA levels in the *mdx* samples are expressed as fold change compared to those in the wt samples. All values represent the mean ± SD of four experiments performed on different RNA preparations of the muscle tissues from wt and *mdx* mice (see Method). Statistical significance was determined as *P<0,05 (*P* = 0,012 as measured by the Mann-Whitney test). B: Total protein extracts from the gastrocnemius muscles of wt and *mdx* mice were resolved by SDS-PAGE and probed with β1-syntrophin antibodies. A representative western blot is shown. The graph values represent the mean ± SD of the densitometric analyses from four independent experiments with different animal samples (see Methods). Data are presented as the percentage of protein in *mdx* mice compared to that in wt mice, normalized to endogenous actin expression level. *P<0,05 versus wt (*P* = 0,002 as measured by the Mann-Whitney test). C: Sections of the gastrocnemius muscles of wt and *mdx* adult mice were probed with an anti-β1-syntrophin antibody and visualized using a secondary antibody coupled to a fluorescent marker, FITC. A representative of the two performed analyses on different animals is shown.

The mRNA level of β1-syntrophin in the muscles of adult *mdx* mice was almost two-fold higher than that in the gastrocnemius muscles of wt animals (Fig. 3A). Shown by western blot analysis, a significant reduction in its protein level (approximately 40%), was observed in the muscles of the adult dystrophic mice (Fig. 3B). Similarly, a reduction of β1-syntrophin protein level was observed when the protein was immunoprecipitated from the whole tissue homogenates of *mdx* muscles compared to those of wt muscles (Fig. S2). As expected, immunofluorescence analysis showed the absence of β1-syntrophin in the plasma membrane of dystrophic skeletal muscle fibers (Fig. 3C).

The same results were obtained when other types of muscles, such as diaphragm, and extensor digitorum longus were investigated (data not shown), suggesting that a reduction in β1-syntrophin protein level is a general phenomenon.

These data showed that the levels of dystroglycans and α-syntrophin were not significantly modified in dystrophic muscles. In contrast, the level of β1-syntrophin was clearly decreased, and this reduction in *mdx* muscles could not be explained by a decrease in gene transcription. Therefore, our studies focused on post-transcriptional mechanisms implicated in the regulation of β1-syntrophin.

### 3′-UTR analysis

The 3′-untranslated regulatory regions (3′-UTRs) of mRNAs play an important role in the regulation of mRNA translation. Therefore, we investigated whether the expression of β1-syntrophin was differentially regulated in skeletal muscles between *mdx* mice and wt mice, and whether this phenomenon was dependent on the 3′-UTR of β1-syntrophin. To this end, the 3′-UTR of the mRNA encoding β1-syntrophin was cloned into the pEGPF-C1 expression vector, allowing us to assess 3′-UTR regulation by examining GFP expression. First, we verified that the construct pEGFP-3′-UTR-β1-synt was expressed in transiently transfected COS1 cells, and that introduction of the 3′-UTR of β1-syntrophin into the plasmid did not constitutively induce GFP expression (data not shown).

**Figure 4:**
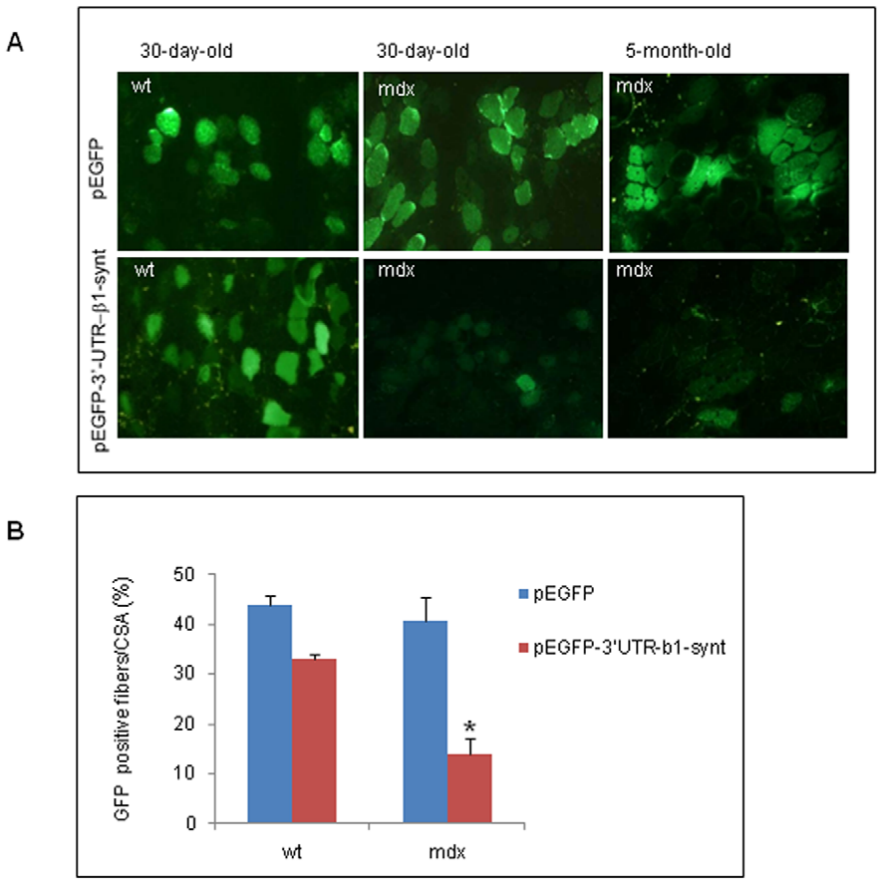
Gene delivery into tibialis muscles of wt and *mdx* mice. A: Micrographs of GFP expression in the muscle sections from tibialis muscles of wt or *mdx* mice injected with 20 µg of DNA containing either pEGPF-C1 or pEGFP-3′-UTR-β1-synt, and subjected to electric pulses. A representative experiment is shown. B: The graph values represent the mean ± SE of the percentage of GFP positive cells/muscle sections analyzed in 30-day old wt and *mdx* mice. Fifteen sections per experimental group (n = 3 wt, n = 4 *mdx*) were counted. *P<0,05 as measured by the unpaired t-test.

To test the hypothesis that the 3′-UTR of β1-syntrophin is differentially regulated in the muscles of dystrophic animals, the pEGFP-3′-UTR-β1-synt construct was electroporated into the tibialis anterior muscle of wt and *mdx* mice. The electroporation procedure induced minimal tissue damage, evaluated by histological observation of the electroporated muscles (data not shown). The results revealed that GFP was consistently detectable in muscle fibers when the empty vector pEGPF-C1, or the pEGFP-3′-UTR-β1-synt construct were transfected into the muscles of wt mice (Fig. 4A), indicating that the 3′-UTR of β1-syntrophin does not interfere with GFP expression in skeletal muscles of wt animals. GFP expression was clearly detected in the muscle of *mdx* mice electroporated with pEGPF-C1; however, the expression was markedly reduced in the muscles *of mdx* mice electroporated with pEGFP-3′UTR-β1-synt (Fig. 4B).

These data demonstrated that, in the presence of the β1-syntrophin 3-′UTR, GFP expression was impaired in the muscles of *mdx* mice, suggesting a differential *in vivo* regulation of 3′-UTR-β1-synt between dystrophic muscles and wt muscles.

### MicroRNA expression

MicroRNAs are well known regulators of protein expression and their action is mediated by binding to the 3'-UTR of target mRNAs. Since we have just shown that the β1-syntrophin 3'-UTR is negatively regulated in dystrophic muscles, we considered the potential role of microRNAs in the modulation of β1-syntrophin expression. No data are currently available about which specific miRs modulate the expression of DAPC proteins; for this reason, to identify putative miR binding sites on the β1-syntrophin 3′-UTR, we took advantage of many computational programs available to predict miRNA targets. We used the target prediction programs, the Miranda package and targetscan, which use microRNAs complementarity to predict target mRNAs. Among the several miRs predicted to bind the β1-syntrophin 3′-UTR, three miRs (miR-222, miR-24, and miR-339) were identified by the different databases utilized.

As the first step, we determined whether the three selected miRs were expressed in myogenic cells, such as primary mouse satellite cells and C_2_C_12_ cell lines, and in skeletal and heart muscle tissues obtained from five-month-old wt mice. Northern blot analysis revealed that all the three miRs were expressed in pooled samples of skeletal muscle tissues, as well as in proliferating C_2_C_12_ myoblasts and differentiated myotubes (data not shown). Next, we investigated whether the differential expression of these miRs occurred in the muscles of *mdx* mice compared to those of wt animals. RNA was prepared from wt and *mdx* skeletal muscle tissues at different ages (20-day, 30-day and five-month-old mice) to follow the disease progression.

**Figure 5:**
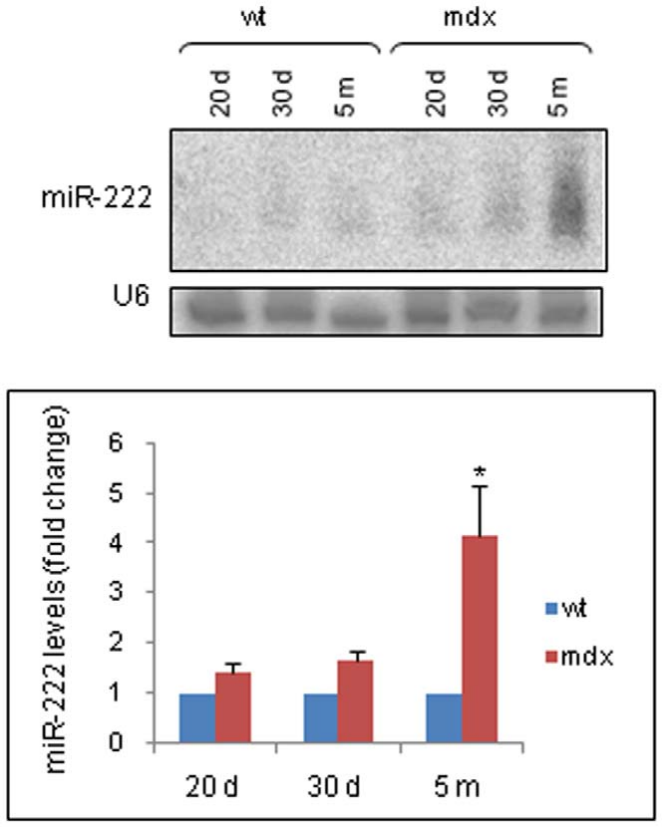
miR-222 expression levels in wt and dystrophic skeletal muscle tissues. A: MiR expression was evaluated by northern blot analysis in the gastrocnemius muscle tissues from wt and *mdx* mice of different ages; U6 levels were used as loading controls. A representative blot from four experiments is shown. B: Northern blot signals were quantified by densitometric analysis. The graph shows the mean ± SD from four independent experiments. For each experiment, pooled muscle tissues were used (see Methods). The miR-222 levels in dystrophic samples are expressed as fold change relative to those in control samples. Statistical significance was determined as *P<0,05 versus wt (*P* = 0,024 measured by the Mann-Whitney test).

Northern blot analysis showed no significant differences in the expression of miR-24 and miR-339 between normal and dystrophic mRNA samples (data not shown). MiR-222 expression was significantly elevated in the gastrocnemius muscles from *mdx* mice compared to those from wt mice. In particular, a 50% increase in miR-222 levels was observed in the muscles of 20- and 30-day-old *mdx* mice, and a maximum of three-fold increase was evident in the muscles of five-month-old *mdx* animals (Fig. 5). Thus, these results demonstrate that miR-222 is expressed in skeletal muscles, and notably upregulated in *mdx* mice, suggesting a potential role of miR-222 in downregulating β1-syntrophin expression in dystrophic muscles.

### Analysis of miR-222 responsive elements within the 3′-UTR of β1-syntrophin

**Figure 6:**
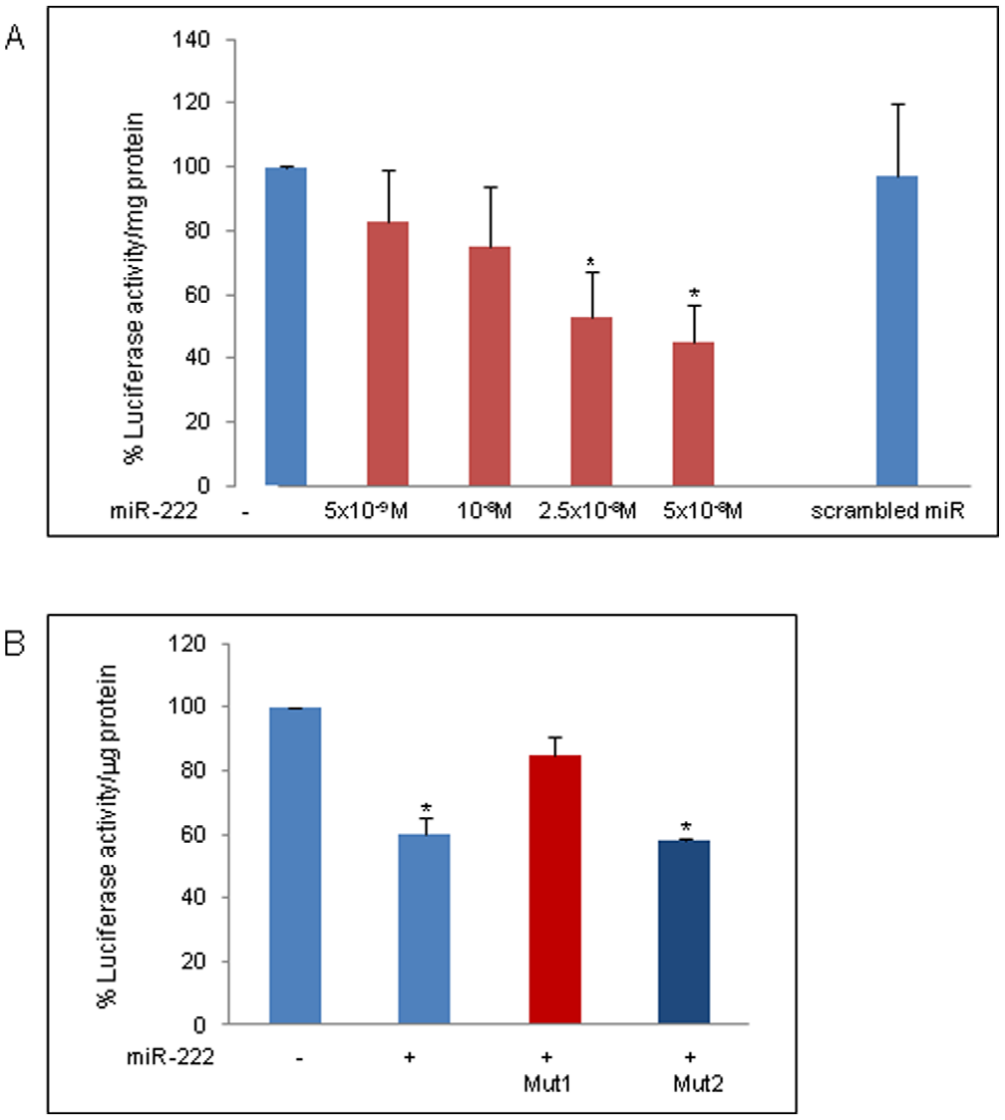
Luciferase activity in the presence of miR-222. A: Luciferase activity in COS1 cells transfected with pGL3-3′-UTR-β1-syntrophin was assessed in the absence or presence of different concentrations of miR-222 and or antimiR (5×10^−8^M). Luciferase activity was normalized to total protein levels and calculated as the percentage of the values obtained from miR-transfected samples compared to those obtained from untreated cells. Results are presented as the mean ± SE from five independent experiments. Statistical significance was determined as *P<0,05 versus untreated cells (*P* = 0,0045 [2.5×10^−8^M], *P* = 0,0027 [5×10^−8^M] measured by the Mann-Whitney test). B: Luciferase activity of COS1 cells was assessed in cells transfected with pGL3-3′UTR-β1-syntrophin or two vectors with mutations in the first (Mut1) or second (Mut2) putative binding site for miR-222 in the absence or presence of 5×10^−8^M of miR-222. Results are presented as the mean ± SD from three independent experiments. *P<0,05 versus untreated cells (*P* = 0,040 measured by the Mann-Whitney test).

To determine whether the β1-syntrophin mRNA is the real target of miR-222, the activity of a luciferase reporter construct containing the 3′-UTR of β1-syntrophin RNA was evaluated in the presence of miR-222. To perform this experiment, the 3′-UTR of β1-syntrophin was cloned into the pGL3 vector, and the construct was transiently transfected into COS1 cells cultured in the absence or presence of different concentrations of miR-222. At the end of the incubation time, the cells were lysed and processed to measure the luciferase activity of the synthesized enzyme. The results showed that luciferase activity was dose-dependently decreased by miR-222 and no luciferase activity was detected when a “scrambled” negative control or an anti-miR specific to miR-222 was used in its place (Fig. 6A). The highest concentration of miR-222 employed (5×10^−8^M) caused a decrease of 50% in luciferase activity, in agreement with the inhibitory effect observed in other cellular systems by the same miR [35], [36]. This result strongly suggested an interaction between miR-222 and the 3′-UTR of β1-syntrophin.

The 3′-UTR of β1-syntrophin contains two putative consensus sites for miR-222 binding. To confirm the target specificity of miR-222, two pGL3-3′UTR β1-syntrophin constructs, carrying deletions of the complementary sequences that could potentially be involved in the binding of miR-222, were used. Luciferase activity was measured in the cells transfected with the vector mutated in binding site 1 (mut1), or in binding site 2 (mut2), in the presence of 5×10^−8^M of miR-222. As shown in Fig. 6B, a mutation of binding site 1 almost completely abolished the activity of miR-222, whereas a mutation of binding site 2 only slightly reduced miR-222 activity, indicating that the majority of miR-222 activity is due to the binding to the first consensus sequence.

### Functional analysis

**Figure 7:**
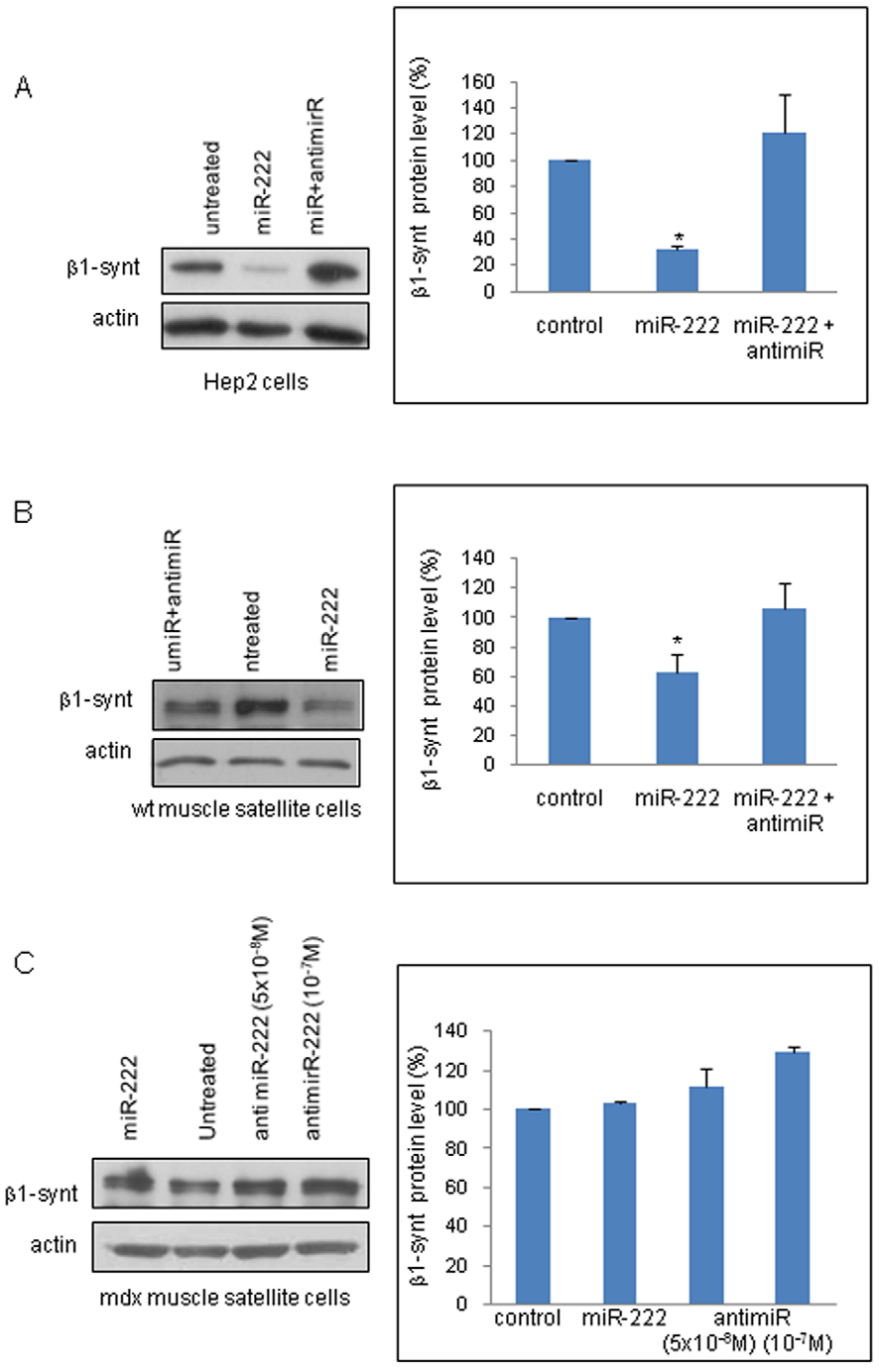
miR-222 modulation of β1-syntrophin levels. Hep2 cells (A) and, muscle satellite cells from wt mice (B) or from *mdx* mice (C) were transfected with miR-222 and/or antimiR-222. Total protein extracts were prepared and analyzed by western blot to assess β1-syntrophin levels. At least three experiments were performed and representative experiments are shown. The graph values represent the mean ± SD of the densitometric analyses from independent experiments (n = 3 for Hep2 cells and *mdx* satellite cells; n = 4 for wt satellite cells). The data are presented as the percentage of protein in treated cells compared to that in untreated cells, normalized to endogenous actin expression level. Statistical significance was determined as *P<0,05 versus untreated cells, measured by the Mann-Withney test (Hep cells, *P* = 0,019; wt satellite cells, *P* = 0,036).

As shown by the luciferase activity assays, miR-222 efficiently targeted the 3′-UTR of β1-syntrophin. To address the role of miR-222 in regulating β1-syntrophin expression, we investigated the effect of overexpressing or silencing miR-222 on endogenous β1-syntrophin levels in cells. β1-syntrophin is expressed at different levels in several tissues, with the highest expression level observed in the liver [37]. Therefore, to obtain a significant signal, the hepatic cell line, Hep2, was used to assess β1-syntrophin modulation in the presence of exogenous miR-222. The Hep2 cells were transfected with miR-222 and/or anti-miR-222, and β1-syntrophin protein levels were determined by western blot. The results showed that β1-syntrophin expression was reduced by approximately 60% in the hepatic cells transfected with miR-222, and its expression was restored in the cells co-transfected with miR-222 and anti-miR-222 (Fig. 7A). This result indicates that exogenous miR-222 reduces the protein level of endogenous β1 syntrophin, regardless of the cell origin.

The same experiment was performed in satellite myogenic cells from the muscles of wt and *mdx* mice. When miR-222 was overexpressed in satellite cells from wt mice, a 40% reduction in β1-syntrophin level was observed; this reduction was abolished when miR-222 activity was blocked by the corresponding antagomir (Fig. 7B). In the satellite cells from dystrophic muscles of young mice, the expression level of miR-222, measured by qRT-PCR, was 50% higher than that in wt cells (data not shown). No reduction in β1-syntrophin level in response to exogenous miR-222 was observed in these cells, whereas treatment with anti-miR-222 led to a slight increase in β1 syntrophin level, thus counteracting the elevated endogenous level of miR-222 in the *mdx* muscles (Fig. 7C). Taken together, these data demonstrate that miR-222 regulates β1-syntrophin protein expression, and silencing miR-222 can restore β1-syntrophin protein expression to normal in dystrophic cells in culture.

Another potential role of miR-222 could be the modulation of other miRs specifically expressed in skeletal muscles. Thus, we investigated whether miR-222 is involved in the expression of three myogenic miRs: miR-1, miR-133 and miR-206. To this end, C_2_C_12_ cells were transfected with miR-222 or anti-miR-222. RNA was then extracted and the expression levels of these myogenic miRs were measured by qRT-PCR. The obtained results (Fig. S3) showed that miR-222 regulated the expression of these myogenic miRs: miR-222 overexpression reduced the expression of all these three miRs. Conversely, anti-miR-222 treatment that inhibited endogenous miR-222 exerted no effect on the expression of miR-1, miR-133 and miR-206, probably due to the low miR-222 expression of in C_2_C_12_ cells.

## Discussion

In this study, we investigated the possible mechanisms regulating the expression of the dystrophin-associated protein complex during the progression of muscular dystrophy. Dystrophin deficiency causes a disruption of the DAPC, with a drastic reduction or loss of its components, and the absence of these proteins in the plasma membrane of muscle cells [14]–[19]. The presence of detectable levels of mRNA for the different protein components of the DAPC, and the absence of the corresponding proteins in the muscles of dystrophic mice, suggest the possible involvement of a post-transcriptional mechanism of regulation. While previous papers [20]–[22] documented the participation of the proteasome degradation system, our study shows that other mechanisms are involved in the regulation of DAPC, particularly, the microRNA system plays an important role by modulating the translation of the mRNA of some DAPC components.

MiRs are involved in several physiological systems including, skeletal muscles [26]–[32], as well as in numerous diseases. It has recently been reported that miRs are differentially modulated in DMD, suggesting an important role of miRs in this disease [28]–[32]. To address the potential mechanistic role of miRs in DMD pathogenesis, we investigated whether miRs are involved in the impaired expression of DAPC components during muscular dystrophy. To this end, we used a well-established animal model of DMD, *mdx* mice. These mice carry a mutation that results in the absence of dystrophin and, subsequently, leads to a reduction of other DAPC components. In *mdx* mice, muscle pathology starts at two weeks of age and is characterized by a continuous cycle of degeneration and regeneration of skeletal muscle cells that peaks between three and four weeks of age. After this phase, the disease becomes milder and is characterized by increasing muscular fibrosis that lasts until the late phase of the animals life [38]. Based on this progression pattern of the disease, we analyzed the RNA and protein expression levels of some DAPC components at different stages of the disease. Our results showed that in *mdx* muscles, the protein level of β1-syntrophin was reduced compared to that in muscles from 30-day- and five-month-old wt mice, whereas, mRNA level of β1-syntrophin increased by 50%. The elevated mRNA level excluded the possibility that a decrease in gene expression was responsible for the protein loss. On the contrary, there was no significant difference in protein and mRNA levels of α-syntrophin, and α- and β-dystroglycan between normal and dystrophic muscles; however, all these proteins were clearly absent in the cell membrane of dystrophic muscles, suggesting that this absence is not due to a block in mRNA translation but more likely due to impaired localization.

Based on these results, our studies focused on the potential post-transcriptional mechanisms that might be involved in β1-syntrophin regulation in dystrophic muscle tissues. In *vivo* experiments confirmed that the absence of β1-syntrophin in muscles from *mdx* mice was due to a block in translation. After pEGFP-3′-UTR-β1-synt was electroporated into the posterior limb muscle of wt and *mdx* mice, histological analysis revealed that GFP was clearly expressed in wt muscles, but only at a very low level in *mdx* muscles, demonstrating a specific modulation of the 3′-UTR. By analyzing this 3′-UTR, we identified putative consensus binding sites for three miRs: miR-24, miR-222 and miR-339. All of these miRs were expressed in skeletal muscle tissues of wt and dystrophic mice. MiR-222 level was higher in dystrophic muscles compared to that in wt muscles, and the levels increased with the progression of the disease. MiR-222 was also the only miR specifically binding the 3′-UTR of the β1-syntrophin mRNA. MiR-222 expression was also detectable in primary satellite cell cultures and was 50% higher in myogenic cells from young *mdx* mice compared to that in wt cells.

Our data are in agreement with previous reports. In fact, miR-222 has been found to be upregulated in 10 different myopathies: a study by Eisenberg *et al.* [31] reported that a large number of miRs were differentially expressed in various muscular pathologies and, that, in particular, the expression of five miRs (miR-146b, miR-221, miR-155, miR-214, miR-222) was altered in all the analyzed syndromes. Recently, Greco *et al.* [32], investigated miR expression in the adductor muscles from *mdx* mice and DMD bioptic samples, and miR-222 was among the eleven dysregulated miRs identified in that study. Together, these data indicated a potential role of miRs in dystrophy and our data confirmed the miR-222 upregulation in muscular dystrophy.

In addition, in agreement with a recent report by Cardinali *et al.* [39], we also detected a reduction in miR-222 expression during myogenic differentiation of C_2_C_12_ cells. These authors observed that in quails, murine primary muscle cells, and myogenic rodent cell lines, the miR-221/miR-222 cluster was highly expressed in proliferating myoblasts but downregulated in differentiated myotubes. The decreased expression of miR-222 during C_2_C_12_ differentiation could explain the corresponding two- to three-fold increase in β1-syntrophin levels in differentiating versus proliferating C_2_C_12_ cells [40]. Cardinali *et al.* showed that p27 acts as a direct target of miR-222, thus implying a prominent role of this miR in the myogenic process. In our study, we showed, for the first time, that the β1-syntrophin protein is a target of miR-222. Furthermore, overexpression of miR-222 in proliferating C_2_C_12_ myogenic cells, induced a modulation of myogenic miRs suggesting a potential novel regulatory role of miR-222 in the differentiation process.

We demonstrated that miR-222 modulates the β1-syntrophin protein in the cells of different tissues. In muscle cells and the hepatic cell line Hep2, it was shown that endogenous β1-syntrophin protein levels were reduced after transfection with miR-222 and that the inhibitory effect of miR-222 was blocked in the presence of anti-miR-222, which then restored β1-syntrophin protein expression to normal. In satellite dystrophic cells expressing high levels of miR-222, anti-miR-222 treatment slightly increased β1-syntrophin protein levels. This experiment clearly indicates that the impairment of β1-syntrophin protein expression in dystrophic cells is due to the elevated levels of endogenous miR-222, and that β1-syntrophin protein levels can be restored by anti-miR treatment.

In addition to its structural role, β1-syntrophin may also perform an important cell signaling role. In different tissues, several partners have been identified to bind β1-syntrophin, including the ATP binding cassette transporter A, the α1D adrenergic receptor isoform, the voltage-gated sodium channel Nav1.5 and, the potassium channel Kir2 [41]–[45]. Given its scaffold β1-syntrophin may play an important role in muscle diseases by regulating signaling complex localization. In particular, loss of β1-syntrophin could impair the function of ionic channels at specific sarcolemma regions, such as neuromuscular junctions. Indeed, β1-syntrophin is mainly localized on the sarcolemma of the type IIb-fast twitch muscle fibers, that are preferentially affected in DMD [34], suggesting that the absence of β1-syntrophin could worsen the disease. Thus, a fine regulation of β1-syntrophin protein levels is necessary for it to exert proper signaling and mechanical roles, allowing for the appropriate cell function.

In conclusion, our results showed that in dystrophic muscle increased miR-222 expression lead to the decrease in β1-syntrophin expression by specifically binding to the 3′-UTR of β1-syntrophin. A specific antagonist of miR-222 allow rescued β1-syntrophin protein expression in dystrophic satellite cells. These data demonstrate, for the first time, that miRs are involved in the regulation of the DAPC components in dystrophic muscles. The involvement of miRs in the pathophysiology of muscle diseases suggests that these molecules could serve as potential targets for a complementary novel therapeutic approach, because silencing miRs could enhance the expression of DAPC components.

## Materials and Methods

### Ethics statement

All procedures involving mice were completed in accordance with the Italian National Institute of Health (protocol n. 118/99-A) and the ethical guidelines for animal care of the European Community Council (directive 86/609/ECC).

C57BL/6J mice and *mdx* mice were obtained from the Charles River Laboratories Italia s.r.l. (Calco, Lecco, Italy) and were housed in the animal facility of the Department of Histology and Medical Embryology under a 12-h light-dark schedule at a constant temperature and with food and water ad libitum. Animals were sacrificed by carbon dioxide asphyxiation. To minimize unnecessary suffering, before gene delivery into tibialis muscles, the mice were anesthetized by an intra-peritoneal injection of a solution of 80 to100 mg/kg ketamine (Intravet, Italy) +10 mg/kg xylazine (Sigma-Aldrich, St. Louis, MO, USA).

After the animals were sacrified, skeletal muscle tissues were collected and divided into three pieces: two were frozen in liquid nitrogen for RNA and protein extractions, and one was frozen in liquid nitrogen cooled isopentan, for immunological studies. All samples were stored at −80°C.

### Database analysis

We used the target prediction programs: the Miranda package and targetscan, available at the following website location: http://microrna.sanger.ac.uk; http://www.targetscan.org; http://www.microrna.org.

### RNA preparation and analysis

RNA was isolated from muscle tissue samples of wt and *mdx* mice of different ages. Four different RNA samples were prepared, each from tissue pools obtained by combining muscles from multiple mice (n = 6, 20-day-old, n = 2, 30-day-old, n = 2, five-month-old wt mice; n = 8, 20-day-old, n = 4, 30-day-old, n = 2, five-month-old *mdx* mice). Total RNA was extracted using the Trizol reagent (Invitrogen, Carlsbard, CA, USA) according to the manufacturer's instructions. RNA expression was determined by northern blot analysis and/or qRT-PCR [46]. Northern blot was performed using 30 µg of total RNA that was separated on a 15% denaturing polyacrylamide gel and transferred from the gel to a Hybond-N^+^ membrane (Amersham Biosciences/GE Healthcare, Piscataway, NJ). Blotted membranes were fixed by UV crosslinking and heating at 80°C for 1 h, and processed for hybridization. Hybridization was carried out overnight at 38°C in a hybridization buffer containing 5X SSC [20X SSC: 3M NaCl, 0.3 M NaCitrate], 20 mM Na_2_HPO_4_ pH 7.2, 7% SDS, 50% formamide, 50 µg/ml salmon sperm DNA and 10^6^ cpm/ml of radiolabed probe. The probes were oligonucleotides synthesized on the basis of sequences complementary to the corresponding miR (see Table 1) and labelled at the 5′end with ^32^P-γATP. Membranes were washed in 5X SSC and 0.2% SDS at 37°C and 2X SSC and 0.2% SDS at 37°C and then exposed to a Fuji phosphoimager screen. Detection was performed using a phospho-imager after an overnight exposure. The same filters were then hybridized with a U6 probe to normalize the RNA levels for quantitative analysis with a densitometer.

**Table 1:** primers used in RNA analysis.

*Dag* (cDNA)	5′-GAGGACCAGGCCACCTTTATTAAG-3′ (forward primer)5′-TTAAGGGGGAACATACGGAG-3′ (reverse primer)
α-synt (cDNA)	5′-ATGGCGTCAGGCAGGCGCGC-3′ (forward primer)5′-GCCTCCGTCTGGTCTGCTGC-3′ (reverse primer)
β1- synt (cDNA)	5′-CAGTCCCCTTATG AGAAGCTC-3′ (forward primer)5′-TCAGGCCACGCCCAGTC-3′ (reverse primer)
GAPDH	5′-ATCACTGCCACCCAGAAGACT-3′ (forward primer)5′-CATGCCAGTGAGCTTCCCGTT-3′ (reverse primer)
β1-synt (3′UTR)	5′-ACAGAGGGCGACTTGCCTTTGG-3′ (forward primer)5′-GGAGACAGCATCCAGGGGTGTGG-3′ (reverse primer)
β1-synt (3′UTR mut1)	5′-CAGGACTGTGACATCAGGCAGTGCAACATGCACAGCCAGGTTCAGGTT-3′ (forward primer)5′-AACCTGAACCTGGCTGTGCATGTTGCACTGCCTGATGTCACAGTCCTG-3′ (reverse primer)
β1-synt (3′UTR mut2)	5′-AGCCAGGTTCAGGTTTAAGGAACATCCCAGCCTCCTCTAT-3′ (forward primer)5′-ATAGAGGAGGCTGGGATGTTCCTTAAACCTGAACCTGGCT-3′ (reverse primer)
miR-222	5′-GAGACCCAGTAGCCAGATGTAGCT-3′
U6	5′-TTGCGTGTCATCCTTGCGCAGG-3′
EGFP	5′- CAT GGT CCT GCT GGA GTT CGT G -3′ (forward primer)5′- CAG GGG GAG GTG TGG GAG GT -3′ (reverse primer)

Quantitative real-time RT-PCR was performed using the DyNAmo SYBR Green 2-step qRT-PCR kit (Finnzymes, Kellaranta, Finland) or the TaqMan MicroRNA Assay kit (Ambion/Applied Biosystem, Piscataway, NJ, USA) according to the protocol for use in the Applied Biosystems 7500 Fast Real-Time PCR System. For quantification analysis the comparative threshold cycle (Ct) method was used. The Ct values of each gene were normalized to the Ct value of GAPDH or Sno142 in the same RNA sample. Gene expression levels were evaluated as fold change using the equation 2^−ddCt^.

The primers used are reported in Table 1.

### Vector constructs

The 3′-UTR of β1-syntrophin was synthesized by RT-PCR. The mRNA sequence of β1-syntrophin was retrieved from GenBank (U89997). Based on this sequence, oligonucleotide couples flanking the 3′-UTR were designed, synthesized and used as primers for RT-PCR. The PCR product was cloned into a PCR-TOPO vector (InVitrogen, Carlsbard, CA, USA), sequenced and, subsequently, subcloned into a luciferase reporter plasmid, pGL3basic (Promega, Madison, WI, USA) for luciferase assay. The same 3′-UTR DNA fragment was subcloned into pEGFP-C (Clontech, Takara Bio USA, CA, USA) at the 3′-end of the EGFP construct in which GFP expression is driven by the CMV promoter. Mutations in the 3′-UTR of β1-syntrophin, were introduced using the Quick Change site-directed mutagenesis kit (Stratagene, La Jolla, CA, USA). Oligonucleotide primers for cloning and mutagenesis are listed in Table 1.

### Cell culture, transfection and luciferase assays

Transfection was performed using the Lipofectamine 2000 reagent (InVitrogen; Carlsbard, CA, USA) according to the manufacturer's protocol.

To assess luciferase activity, COS-1 cells (ATCC, CRL-1650) were cultured in DMEM supplemented with 10% fetal calf serum. One day prior to the transfection, the cells were plated onto 12-well plates at a density of 80,000/well. The next day, the cells were co-transfected with 1 µg of the pGL3 constructs, and appropriate amounts of miR (Ambion/Applied Biosystem, Piscataway, NJ, USA) and Lipofectamine 2000. The cells were cultured for 4 h in DMEM without serum or antibiotics. Afterwards, the medium was changed, and the cells were incubated in medium supplemented with serum. After 24 h, the cells were collected and homogenized in 70 µl of lysis buffer (Promega, Madison, WI, USA). The cell lysates were centrifuged for 5 min at 12,000 g. Luciferase activity was detected using the luciferase reporter assay system (Promega) and measured by a luminometer. Protein concentration was determined by the BCA assay (Pierce/Thermo Scientific, Rockford, IL, USA).

For western blot analysis, the cells were plated at a density of 70,000/well and transfected either with or without 5×10^−8^ M miR-222 or antimiR-222 (Ambion/Applied Biosystem, Piscataway, NJ, USA). After an incubation of 4 h in medium without serum or antibiotics, the cells were incubated for another 36 h in medium supplemented with serum. At the end of the incubation time, the cells were collected and homogenized in 70 µl of Laemmli buffer. The following cell lines were used: C_2_C_12_ (ATCC, CRL-1772) and Hep2 (ATCC, CCL-23).

In another series of experiments, C_2_C_12_ cells were transfected with miR-222 or antimiR-222 and the expression levels of miR-1, miR-133, miR-206 were measured. One day prior the transfection, the cells were plated at a density of 10,000/cm^2^. The next day the cells were co-transfected with 5×10^−8^ M miR or antimiR (Ambion/Applied Biosystem, Piscataway, NJ, USA) and Lipofectamine 2000. The cells were cultured for 4 h in DMEM without serum or antibiotics. Afterwards, the medium was changed, and the cells were incubated in medium supplemented with serum. After 24 h, the cells were collected, and RNA was prepared using the Trizol reagent as indicated above.

### Primary cell cultures

Primary cell cultures were performed according to Matthew et al. [47]. Briefly, dissected muscles from five to six one-week-old animals (n = 20 wt and 24 *mdx* mice in total) were minced by a razor blade and then digested by collagenase/dispase. The resulting cellular suspension was plated for a few hours to remove contaminating fibroblasts, and the unattached muscle cells were collected and plated on collagene-coated dishes in DMEM (Gibco/InVitrogen, Carlsbard, CA, USA) supplemented with 10% serum. The cultured cells were later processed for transfection experiments and western blot analysis.

### Protein analyses

Frozen muscle tissue samples from wt and *mdx* mice were homogenized in lysis buffer (2% SDS, 5 mM EDTA) supplemented with proteinase inhibitors, boiled for 5 minutes and centrifuged at 12,000 g for 10 min [48]. Protein concentration was determined by the BCA assay (Pierce/Thermo Scientific, Rockford, IL, USA). For each preparation multiple muscle tissues were pooled (n = 2, 30-day-old and n = 1 wt 5-month-old wt mice; n = 3, 30-day-old and n = 1, 5 month-old *mdx* mice.)

Protein aliquots ranging from 30–40 µg were separated by electrophoresis on a 10% SDS-acrylamide gel and transferred onto a nitrocellulose membrane. The blotted membranes were blocked in TBS-T (20 mM Tris-HCl pH 7.5, 137 mM NaCl, and 0.1% Tween-20) containing 5% nonfat dry milk for 30–60 min at room temperature, and then incubated with specific antibodies diluted in the same buffer, overnight at 4°C. The membranes were washed several times with TBS-T and then probed with secondary antibodies coupled to horseradish peroxidase. The immunoreactive bands were visualized by chemiluminescence (ECL or ECL plus kit, Amersham Biosciences/GE Healthcare, Piscataway, NJ). Densitometric analysis was performed using the AIDA 2.2 image software. The optical density (OD) of each signal was normalized to α-actin. The following antibodies were used: mouse anti-α syntrophin (Sigma-Aldrich, St. Louis, MO, USA), goat anti β1-syntrophin (Santa Cruz Biotechnology; Santa Cruz, CA, USA), rabbit anti-β1-syntrophin (antibody SYN37, generously provided by Dr. Stanley Froehner, University of Washington, Seattle WA), mouse anti α-β syntrophins (Affinity Bioreagents, Golden, CO, USA), mouse anti-α and anti-β-dystroglycan (SantaCruz Biotechnology; Santa Cruz, CA, USA), and mouse anti-actin and anti-tubulin (Chemicon/Millipore, Billerica, MA, USA).

Immunoprecipitation analyses were performed following a standard protocol as previously described [49]. Muscle tissue fragments from young and adult wt and *mdx* mice were lysed in RIPA buffer (50 mM Tris-HCl pH 7.4, 150 mM NaCl, 1 mM EDTA, 1% NP-40, 0.25% deoxycholate, and 0.1% SDS) and aliquots containing 1 mg of protein extracts were incubated overnight at 4°C with an antibody anti α-β-syntrophins (Affinity Bioreagents, Golden, CO, USA). The protein-antibody complex was recovered by incubation with protein G-sepharose, separated on SDS-PAGE and probed with an anti-β1-syntrophin antibody.

To determine the localization of different proteins, immunofluorescence analysis, was performed as documented [50]. 7 µm sections were prepared from frozen muscles, permeabilized with 0.1% Triton-X-100, blocked with 5% BSA-TBS-T and incubated with primary antibodies. The specific proteins were visualized using secondary antibodies coupled to a fluorescent marker (fluorescein isothiocyanate, FITC or Texas red; Pierce, Thermoscientific, Rockford, Il, USA). Immunostained samples were counterstained with DAPI (4′,6′-diamidino-2-phenylindole dihydrochloride: Molecular Probes, Eugene, OR) and examined by a Zeiss Axioskop2 plus microscope**.** Images were obtained with a Zeiss AxioCam HRc using the Axiovision software.

The following antibodies were used: α-dystroglycan clone VIA4-1 (Millipore, Billerica, MA, USA), β-dystroglycan clone 43DAG1/8D5 (Monosan, Uden, NL), α-syntrophin RA16-1 (Sigma-Aldrich, St. Louis, MO, USA), β1-syntrophin clone C20 (SantaCruz Biotechnology, Santa Cruz, CA, USA).

For this analysis, a total of n = 3 wt mice and n = 3 *mdx* adult mice were used.

### Gene delivery into tibialis muscles

In vivo experiments were performed on 30-days-old wt (n = 3) and *mdx* mice (n = 5) or 5-month-old *mdx* mice (n = 2) [51]. Tibialis anterior muscles were exposed using a short incision and 20 µl of a solution of 5% mannitol containing 20 µg of DNA (pEGPF−C1 or pEGFP-3′UTR-β1-synt) was injected using a Hamilton syringe in the central part of the muscle. One leg received the empty vector pEGPF-C1, the other the pEGFP-3′UTR-β1-synt construct. Afterwards, the muscle was separated from tibia, and a pair of spatula-like electrodes (0.5 cm wide, 2 cm long) were placed at each side of the muscle close to the injection site (anode was positioned under the muscle in transverse direction relative to the fibers, and cathode was placed on the muscle in longitudinal direction) and electric pulses were delivered. Six electric pulses with the fixed pulse duration of 50 msec/pulse, were delivered at an interval of 200 ms using an electric pulse generator. The ratio of applied voltage to electrode distance was 5V/mm (muscle thickness). Mice were sacrificed 24 h after the electroporation and muscles were removed, frozen in liquid nitrogen-cooled isopentan, and then processed for morphological analysis.

Several 7-µm thick cryostat sections, upstream and downstream of the injection site were prepared, GFP expression was examined using a Zeiss Axioskop2 plus fluorescence microscope (Carl Zeiss, Oberkochen, Germany). As a control for gene delivery, PCR and RT-PCR analyses were performed on genomic and RNA samples, respectively. Oligonucleotide primers for GFP are listed in Table 1.

### Statistical analysis

Data are presented as mean ± standard deviation (SD) or standard error (SE). Statistical significance between groups was determined using the GraphPad Prism software. The non parametric-Mann-Whitney test was done for the analysis of RNA expression, protein expression and luciferase activity when sample groups were small. The unpaired t-test was performed to assess the significativity of gene delivery experiments. *P* values of less than 0.05 were considered statistically significant.

## Supporting Information

Figure S1 Dystroglycan protein expression. A: Total protein extracts were obtained from the gastrocnemius muscle tissues of wt and *mdx* mice of different ages (30 d, 30-day-old mice; 5 m: five-month-old mice) and were resolved by SDS-PAGE and transferred to nitrocellulose membrane. The membrane was probed with α and β-dystroglycan antibodies. Representative western blots are shown. The graph values represent the mean ± SD of the densitometric analyses from three independent experiments; the data are presented as the percentage of protein in *mdx* mice compared to that in wt mice, normalized to endogenous tubulin expression level. B: The gastrocnemius muscle sections from wt and mdx adult mice were probed with α and β−dag antibodies and visualized using secondary antibody coupled to a fluorescent marker, Texas red or FITC.
(7.35 MB TIF)

Figure S2 β1-syntrophin protein expression. A representative experiment of immunoprecipitation is shown. Whole homogenates obtained by RIPA buffer extraction were immunoprecipitated with an antibody against syntrophins. The immunomplexes were separated on an SDS-PAGE and probed with a β1-syntrophin specific antibody. The experiment was performed twice with similar results.
(0.28 MB TIF)

Figure S3 miR-222 modulation of myogenic miRs expression. RNA levels of miR-206, miR-1, and miR-133 in C_2_C_12_ cells transfected with miR-222 (5×10^−8^M) or anti-miR-222 (5×10^−8^M) were assessed by qRT-PCR; relative gene expression was calculated by the comparative Ct method (2^−ddCt^). The Ct values of each miR were normalized to the Ct value of sno142 in the same RNA samples. RNA levels in cells treated with miR-222 or anti-miR-222, are expressed as fold change compared to those in the untreated cells. A representative of the two performed experiments is shown. All values represent the mean ±+ SD from triplicate samples.
(2.02 MB TIF)
