# Supplementary figures and images for: β1-Syntrophin Modulation by miR-222 in mdx Mice
Source: PLoS One. 2010 Aug 10;5(8):e12098. doi: 10.1371/journal.pone.0012098 (PMC2938373; doi:10.1371/journal.pone.0012098)

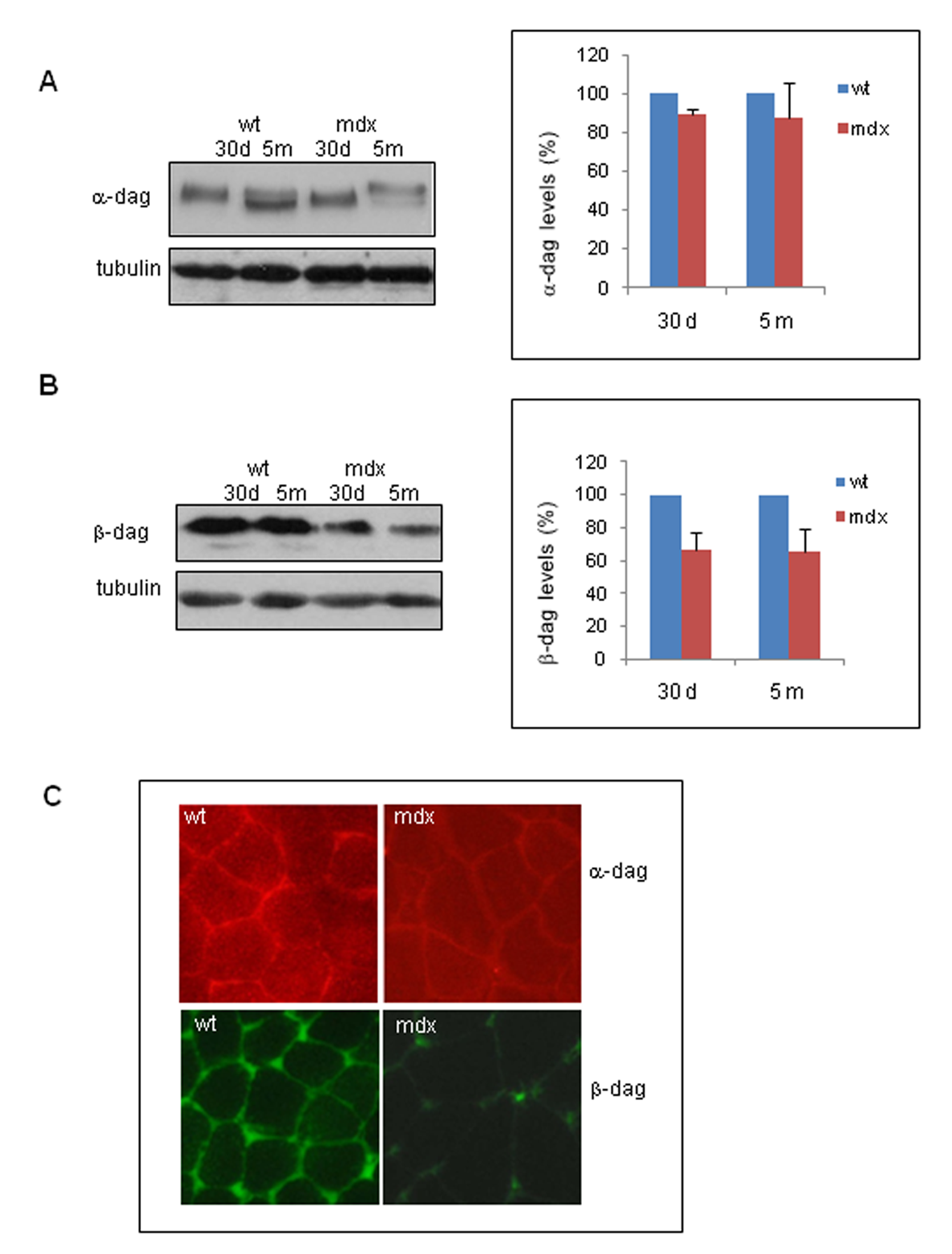

Supplement: Figure S1 — Dystroglycan protein expression. A: Total protein extracts were obtained from the gastrocnemius muscle tissues of wt and mdx mice of different ages (30 d, 30-day-old mice; 5 m: five-month-old mice) and were resolved by SDS-PAGE and transferred to nitrocellulose membrane. The membrane was probed with α and β-dystroglycan antibodies. Representative western blots are shown. The graph values represent the mean ± SD of the densitometric analyses from three independent experiments; the data are presented as the percentage of protein in mdx mice compared to that in wt mice, normalized to endogenous tubulin expression level. B: The gastrocnemius muscle sections from wt and mdx adult mice were probed with α and β−dag antibodies and visualized using secondary antibody coupled to a fluorescent marker, Texas red or FITC. (7.35 MB TIF) [file pone.0012098.s001.tif]

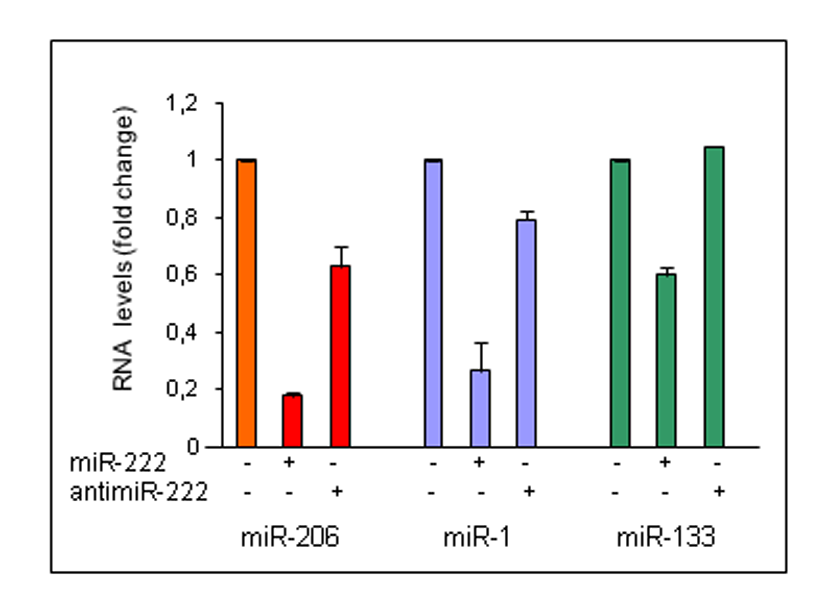

Supplement: Figure S3 — miR-222 modulation of myogenic miRs expression. RNA levels of miR-206, miR-1, and miR-133 in C2C12 cells transfected with miR-222 (5×10^−8^M) or anti-miR-222 (5×10^−8^M) were assessed by qRT-PCR; relative gene expression was calculated by the comparative Ct method (2−ddCt). The Ct values of each miR were normalized to the Ct value of sno142 in the same RNA samples. RNA levels in cells treated with miR-222 or anti-miR-222, are expressed as fold change compared to those in the untreated cells. A representative of the two performed experiments is shown. All values represent the mean ±+ SD from triplicate samples. (2.02 MB TIF) [file pone.0012098.s003.tif]
